# Supplementary figures and images for: The deletion of a major facilitator superfamily gene VdMFS2 results in enhanced pathogenicity of Verticillium dahliae to cotton
Source: Microbiol Spectr. 2026 May 11;14(6):e02761-25. doi: 10.1128/spectrum.02761-25 (PMC13228058; doi:10.1128/spectrum.02761-25)

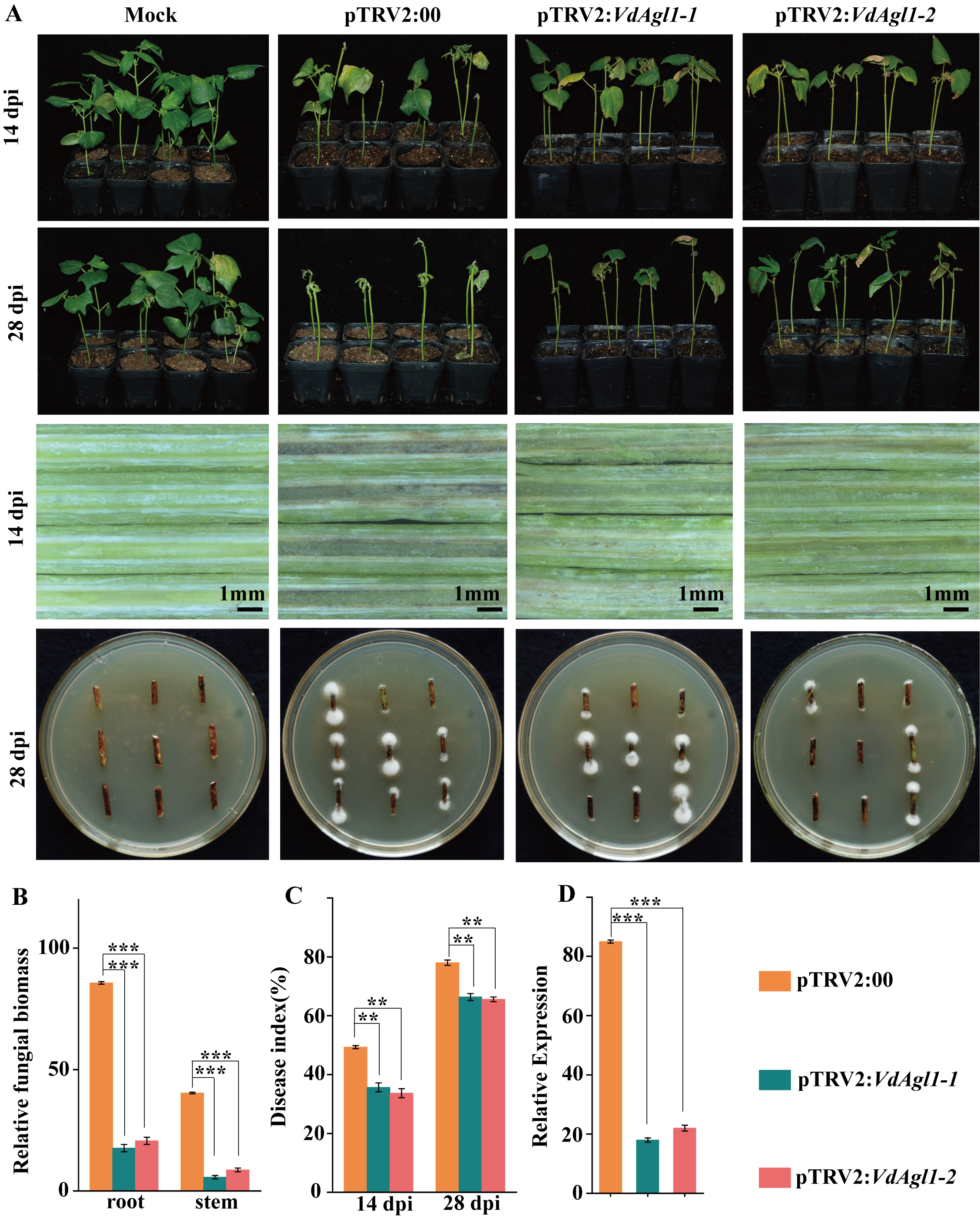

Supplement: Fig. S1A [file spectrum.02761-25-s0001.tif]
