# Supplementary material for: The deletion of a major facilitator superfamily gene VdMFS2 results in enhanced pathogenicity of Verticillium dahliae to cotton
Source: Microbiol Spectr. 2026 May 11;14(6):e02761-25. doi: 10.1128/spectrum.02761-25 (PMC13228058; doi:10.1128/spectrum.02761-25)
Supplement: Fig. S1C — Heatmap showing the expression fold change of N-SCPs in different comparisons. [file spectrum.02761-25-s0003.docx]

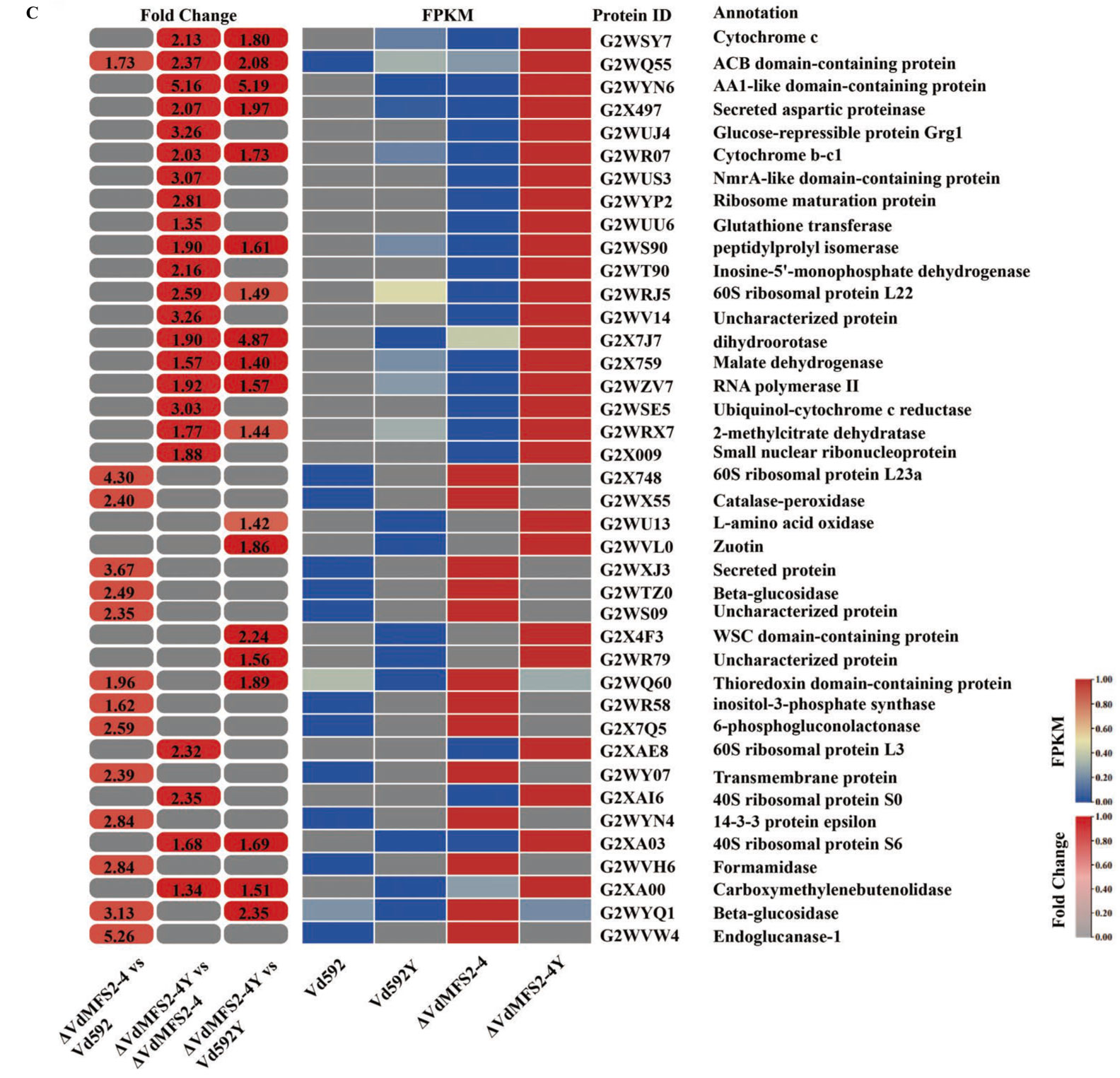
Fig. S1C

**Fig. S1** (C) Heatmap showing the expression fold change of N-SCPs in different comparisons. The heatmap was generated based on the Proteomics data. The numbers in heatmap represent fold change, p<0.05, FC≥1.2.
