# Supplementary material for: The deletion of a major facilitator superfamily gene VdMFS2 results in enhanced pathogenicity of Verticillium dahliae to cotton
Source: Microbiol Spectr. 2026 May 11;14(6):e02761-25. doi: 10.1128/spectrum.02761-25 (PMC13228058; doi:10.1128/spectrum.02761-25)
Supplement: Table S1 — Primer sequences. [file spectrum.02761-25-s0004.docx]

Table S1 Primer sequences

| Primer name | Primer sequence (5′-3′) | Purpose |
| --- | --- | --- |
| HIGS-*VdMFS2*-F | GGAATTCTACGTCGCGTGCTTCATCTT | PCR |
| HIGS-*VdMFS2*-R | GGGTACCCGTAAAGATTGCCGTGGTCG |  |
| *VdMFS2*-Flank-5’F | GGTGGCGGCCGCTCTAGAAACAGTGGCTTTCTTACCGA | PCR |
| *VdMFS2*-Flank-5’R | AAAATGCTCCTTCAAAAGTTACAATGGAGAGACAGGC |  |
| *VdMFS2*-Hyg-F | GCCTGTCTCTCCATTGTAACTTTTGAAGGAGCATTTT | PCR |
| *VdMFS2*-Hyg-R | CCGAAAGCCATTCTACATACAGTTATCTTTGCGAACC |  |
| *VdMFS2*-Flank-3’F | GGTTCGCAAAGATAACTGTATGTAGAATGGCTTTCGG | PCR |
| *VdMFS2*-Flank-3’R | GACGGTATCGATAAGCTTCGTCATCGTCGTCATCTACG |  |
| *VdMFS2-C*-F | ACGGCCAGTGCCAAGCTTTAGACAGATCCAACTTCCCTG | PCR |
| *VdMFS2-C*-R | ATTCACTAGTGGATCCTGCCCAAGGACATCCACGTCA |  |
| PDR-*VdMFS2*-F | GCGGCCGCGCGGATCATGACGACCCTCAGGAGAA | PCR |
| PDR-*VdMFS2*-R | TCCAAAGCTGGATCGTACCCGTAAAGATTGCCGTG |  |
| *VdMFS2*-qPCR-F | GCGTTTGCCTTTGGTTCCAT | qRT-PCR |
| *VdMFS2*-qPCR-R | CGTAAAGATTGCCGTGGTCG |  |
| *β-Tubulin*-F | TCCACCTTCGTCGGTAACTC | qRT-PCR |
| *β-Tubulin*-R | GCCTCCTCCTCGTACTCCTC |  |
| *Ve-ITS1*-F | AAAGTTTTAATGGTTCGCTAAGA | qRT-PCR |
| *ST-VE1*-R | CTTGGTCATTTAGAGGAAGTAA |  |
| *GhUBQ7*-F | GAAGGCATTCCACCTGACCAAC | qRT-PCR |
| *GhUBQ7*-R | CTTGACCTTCTTCTTCTTGTGCTTG |  |
| HIGS-*VdAgl1*-F | AAGGTTACCGAATTCATGCGCGGCCTGATTCTCCTC | PCR |
| HIGS-*VdAgl1*-R | CTCGGTACCGGATCCGATGAGCTCGGGGAGTTCTTC |  |
| *VdAgl1*-qPCR-F | CGCCTCATCTATCAGGCTGG | qRT-PCR |
| *VdAgl1*-qPCR-R | GTAGAGTTGTCCGCCGTCAC |  |
