# Supplementary material for: The deletion of a major facilitator superfamily gene VdMFS2 results in enhanced pathogenicity of Verticillium dahliae to cotton
Source: Microbiol Spectr. 2026 May 11;14(6):e02761-25. doi: 10.1128/spectrum.02761-25 (PMC13228058; doi:10.1128/spectrum.02761-25)
Supplement: Table S2 — The list of PHI proteins identified from 94 SPs. [file spectrum.02761-25-s0005.docx]

| **PHI proteins** | **Related**  **genes** | **Pathogen**  **species** | **Mutant phenotypic characteristic** | **References** |
| --- | --- | --- | --- | --- |
| G2WT22 | *SPE3-LYS9* | *C.neoformans* | Loss of pathogenicity | (87) |
| G2WWV4 | *tldD* | *Y.enterocolitica* | Unaffected pathogenicity | (88) |
| G2X9X6 | *pepN* | *S.pneumoniae* | Reduced virulence | (89) |
| G2WZZ8 | *PoMuk1* | *M.oryzae* | Unaffected pathogenicity | (90) |
| G2X7C8 | *FGSG_12242* | *F.graminearum* | Unaffected pathogenicity | (91) |
| G2WSY7 | *cycA* | *A.fumigatus* | Reduced virulence | (92) |
| G2WQ55 | *Acb1* | *M.oryzae* | Reduced virulence | (93) |
| G2X497 | *Cgyps1* | *C.glabrata* | Reduced virulence | (94) |
| G2WR07 | *leuB* | *X.oryzae* | Reduced virulence | (95) |
| G2WS90 | *Fpr1* | *B.bassiana* | Reduced virulence | (96) |
| G2WT90 | *MoImd4* | *M.oryzae* | Reduced virulence | (97) |
| G2WV14 | *ppk1* | *P.aeruginosa* | Reduced virulence | (98) |
| G2WX55 | *FCA6* | *F.graminearum* | Rnaffected pathogenicity | (99) |
| G2WYN4 | *ARSEF* | *B.bassiana* | Reduced virulence | (100) |

Table S2 The list of PHI proteins identified from 94 SPs
